# Bibliometric analysis of rhein in the treatment of tumors

**DOI:** 10.3389/fonc.2025.1550016

**Published:** 2025-02-06

**Authors:** Lan Jiang, Qian Huang, Zhongquan Shi, Yi Yang

**Affiliations:** ^1^ Chongqing Three Gorges Medical College, Chongqing, China; ^2^ Chongqing Engineering Research Center of Antitumor Natural Drugs, Chongqing, China; ^3^ Chongqing Key Laboratory of Development and Utilization of Genuine Medicinal Materials in Three Gorges Reservoir Area, Chongqing, China; ^4^ Department of Pharmacy, Chongqing University Three Gorges Hospital, Chongqing, China

**Keywords:** rhein, cancer, bibliometric analysis, knowledge graph, CiteSpace

## Abstract

**Background:**

Rhein is an anthraquinone compound with a variety of biological activities. It has inhibitory effects on liver cancer, breast cancer, lung cancer, oral cancer, gastric cancer, and other cancers. However, a comprehensive bibliometric review of this field has not yet been published.

**Objective:**

This study aims to investigate and evaluate the current research trends and directions about the antitumor properties of rhein using bibliometric analysis.

**Methods:**

The literature related to rhein in cancer treatment from 2003 to 2023 was retrieved from the Web of Science Core Collection (WoSCC) database, and the annual number of publications, main authors, major institutions, keyword clustering, and keyword bursts were visually analyzed using CiteSpace 6.3.R1 software. This study aims to discuss the status quo, hotspots, and development trends of rhein research over the past 20 years.

**Results:**

A total of 220 articles were retrieved from the core collection database, and the number of articles related to treating tumors with rhein increased annually. Among them, Chung, Jing Gung has the highest number of articles in this field, but most researchers lack cooperation with each other. The institutions with the highest number of articles were the Nanjing University of Chinese Medicine (13 articles) and China Medical University (Taiwan) (13 articles). Research hotspots include the promotion of apoptosis, endoplasmic reticulum stress, inhibition of proliferation, drug resistance, and nanoparticles.

**Conclusion:**

Rhein exerts antitumor effects by inducing cell apoptosis, controlling metastasis, and inhibiting proliferation. However, owing to its poor water solubility, in recent years, functional modification of its functional groups or production of rhein nanoparticles to enhance its bioavailability and antitumor effects has become a hot research direction in the future.

## 1 Introduction

Unquestionably, one of the most important health issues of our day is cancer, a terrible illness that claims millions of lives globally. In addition to causing patients and their families’ great physical and psychological anguish, it also places a heavy financial strain on society all over the world. In February 2024, the World Health Organization’s International Agency for Research on Cancer (IARC) released its latest report on global cancer data. In 2022, the number of new cancer cases worldwide reached 20 million, with deaths reaching 9.7 million (1, 2). The development of cancer is a long process. During this period, normal cells accumulate genetic mutations, which lead to uncontrolled growth, infiltration, and metastasis to various organ systems (3). Over the past few decades, a wide range of studies have developed different treatment modalities such as surgery, radiotherapy, chemotherapy, targeted therapy, and immunotherapy (4–6). Until now, chemotherapy has been the main treatment method. However, owing to its poor water solubility, low bioavailability, and general biological diffusion as well as drug resistance, chemotherapy cannot be widely used. Therefore, there is an urgent need for more effective and less toxic anticancer treatments and drugs (7, 8).

**Figure 1:**
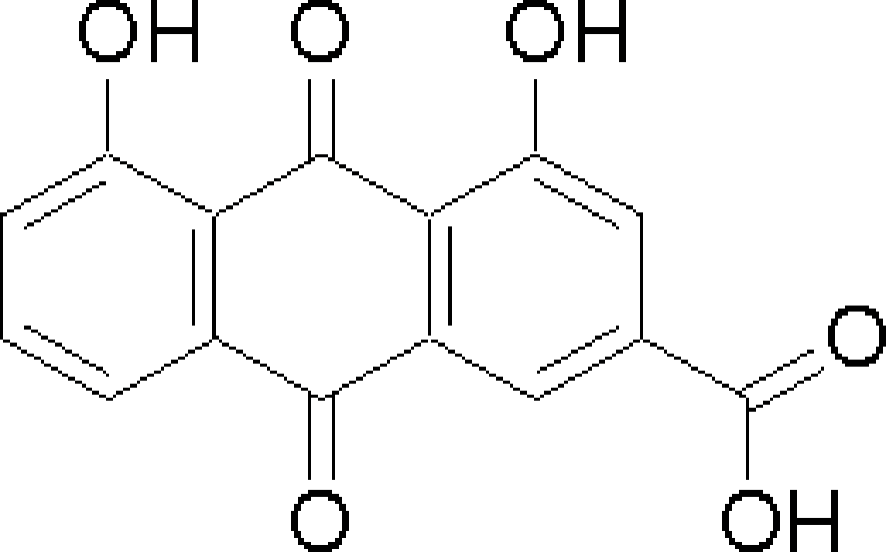
Structural formula of Rhein.

Rhein is an anthraquinone compound with a variety of biological activities, mainly found in the rhizomes of *Rheum palmatum*, He Shou Wu, and other plants. Its chemical formula is C_15_H_8_O_6_, and the structural formula is shown in **Figure 1** (9–11). It has antibacterial, diuretic, laxative, anti-inflammatory, and antidiabetic effects (12–16). In addition, it has demonstrated anticancer activities against a variety of cancers such as liver cancer, breast cancer, lung cancer, oral cancer, and gastric cancer by inhibiting cell proliferation, inducing apoptosis, preventing metastasis, and immunomodulation (17–23). In this study, we mainly analyzed the data with the help of CiteSpace software, which is a literature analysis tool developed by Prof. Chaomei Chen, a Chinese American, and can be used to explore the evolution of scientific knowledge and to uncover hotspots and future trends in the field (24, 25). In the field of bibliometrics, CiteSpace is generally regarded as the most authoritative, scientific, and influential bibliometric software (26, 27). So far, CiteSpace has been used by scholars at home and abroad in many fields, including research management, academic evaluation, and scientific research program management (28–30). In this study, CiteSpace software was used to conduct a comprehensive visualization and analysis of the literature on the treatment of tumors with rhein in the core database of Web of Science over the past two decades to explore the current status, hotspots, and trends of research on the treatment of tumors with rhein, and to provide a certain theoretical reference value for the development and utilization of rhein.

## 2 Materials and methods

### 2.1 Data collection and processing

The Web of Science core database contains records of papers from the world’s most influential journals, conference proceedings, and books. Compared with other relevant databases, information obtained from the Web of Science (WoS) is considered more indicative and impactful. Furthermore, it was shown that more efficient visualization could be achieved utilizing WoS data for bibliometric analyses (31, 32).

The following search terms were employed to retrieve literature from WoSCC: TS=(“rhein” AND (“tumor” OR “tumors” OR “cancer” OR “cancers”)), and the search period was 2003-2023. A total of 220 relevant papers were retrieved.

### 2.2 Research methodology

The 220 core collection documents retrieved were exported in plain text format, and the contents of the records were selected as “full records and cited references”. Visual analysis was performed using CiteSpace software (V6.3.R1) software. The metrics examined included the annual publication count, national and institutional contributions, author co-occurrence, keyword co-occurrence, and keywords with citation bursts in this research field. The period was set from 2003 to 2023, the time slice was set to 1 year, and graph pruning methods were chosen as the pathfinder and pruning sliced networks. The size of the circle in the graph represents the frequency. The larger the diameter, the higher the node frequency.

## 3 Results and analysis

### 3.1 Annual number of publications

**Figure 2:**
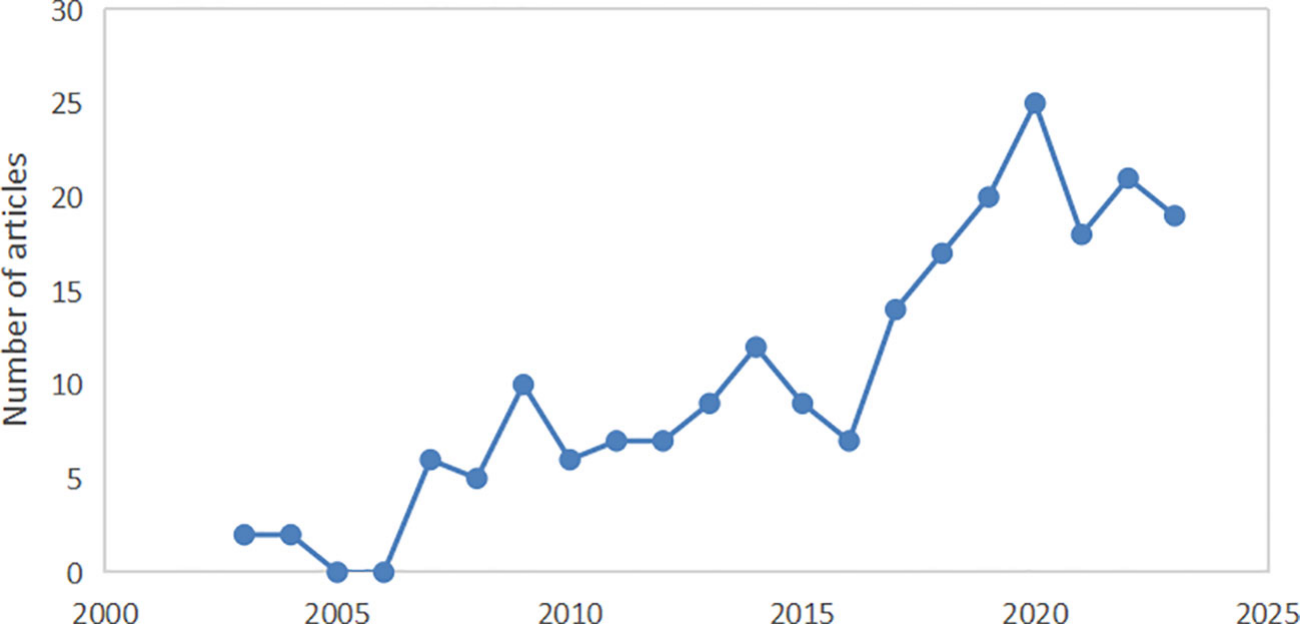
Annual publications of thesis.

The annual publication volume indicator reflects the research situation and trends in a particular field. The 220 retrieved documents were analyzed econometrically to obtain the trend of annual publication data over the past two decades. The results are shown in **Figure 2**, where the vertical and horizontal coordinates indicate the annual publication volume and publication time, respectively. Few studies have been conducted on the treatment of tumors with rhein before 2006, and there has been a gradual increase in the number of publications annually. The annual number of publications generally showed an increasing trend, indicating that attention to the role of rhein in tumors has been increasing annually. By 2020, the number of articles reached a maximum of 25.

### 3.2 Analysis of published journals

A journal’s publication status reflects its degree of focus and influence in the field. Statistics from the literature were used to draw a statistical table of the top five journals in terms of the number of publications on rhein for the treatment of tumors (**Table 1**), indicating that these journals are important in the field of tumor therapy and drug research. Among them, JOURNAL OF ETHNOPHARMACOLOGY has the highest number of articles; it is an SCI medical zone 1 journal, and its main areas of interest include the use of plants, fungi, animals, microorganisms, and minerals and their pharmacological effects, with a special focus on ethnomedicines such as Chinese herbs.

**Table 1:** Statistical table of periodicals.

Serial number	Periodical	Number of articles
1	JOURNAL OF ETHNOPHARMACOLOGY	11
2	BIOMEDICINE PHARMACOTHERAPY	5
3	INTERNATIONAL JOURNAL OF ONCOLOGY	5
4	PHYTOMEDICINE	5
5	AMERICAN JOURNAL OF CHINESE MEDICINE	4

### 3.3 Author network analysis

**Figure 3:**
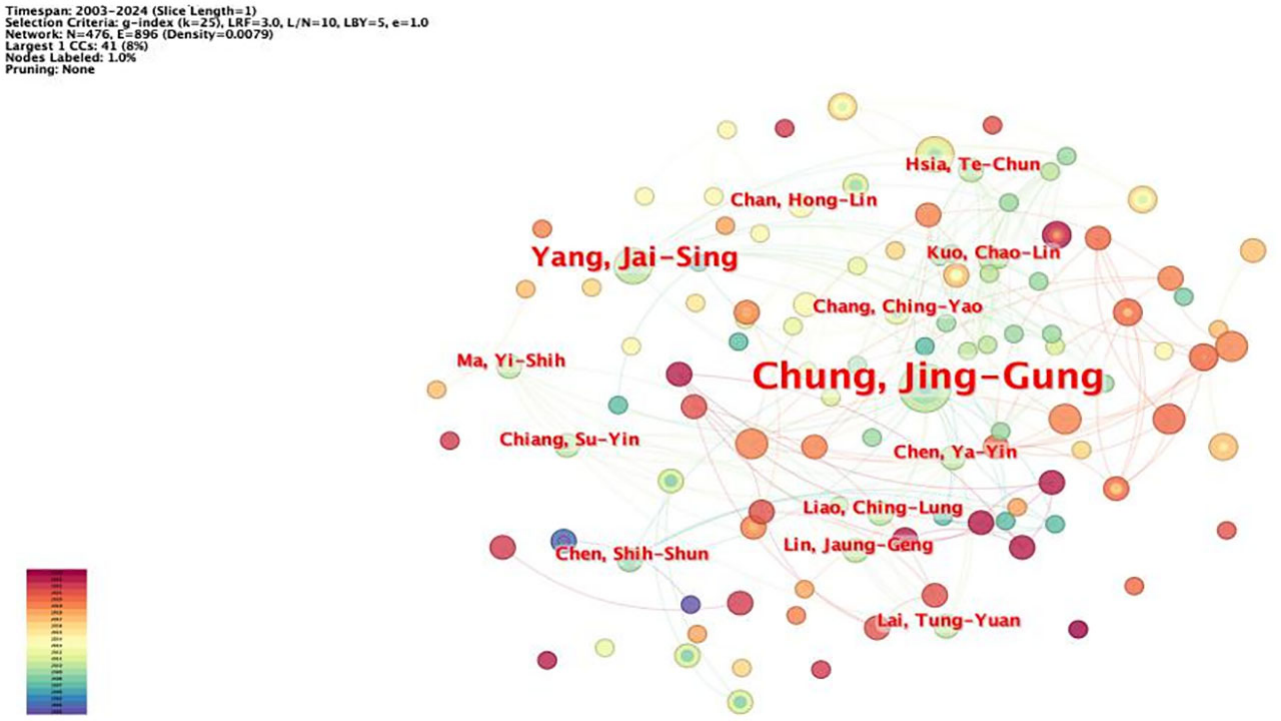
Author co-occurrence mapping.

The network node type was set as “Author,” and the software was used to draw the author network map (**Figure 3**), which has 476 nodes, 896 lines, and a network density value of 0.0079, which means that there are 476 authors and 896 lines of inter-author cooperation. The statistics of authors with more than five publications (**Table 2**), among which Prof. Chung, Jing-Gung from China Medical University (Taiwan), has the most publications (eight articles), were plotted. This research team mainly studied the mechanism underlying the inhibitory effect of rhein on the tongue, nasopharyngeal carcinoma, lung carcinoma, and other tumors. It was found that rhein could play an antitumor effect by inducing cell cycle blockage and apoptosis, reducing the expression of matrix metalloproteinase-9 (MMP-9) and vascular endothelial growth factor (VEGF) to inhibit invasion, etc. (33, 34) The team of Wu, Li from Nanjing University of Traditional Chinese Medicine (NJUTM) is in second place (seven articles), focusing on the synergistic antitumor activity of rhein and doxorubicin (DOX), combining rhein with DOX to make highly efficient nanomaterials, which improves the bioavailability of rhein and at the same time reduces the resistance to chemotherapeutic drugs (35–37). From author mapping, low author centrality indicates that there is less collaboration between authors and that researchers in this research area have not formed extensive collaborations.

**Table 2:** Volume of publications by authors.

Serial number	Author	Author’s unit	Number of articles
1	Chung, Jing-Gung	China Medical University Taiwan	8
2	Wu, Li	Nanjing University of Chinese Medicine Coll Pharm	7
3	Lin, Yajun	Sanmen Dist Ctr Dis Control & Prevent, SANMEN	7
4	Li, Danrong	Guangxi Medical University Life Sci Inst	6
5	Zhen, Yongzhan	North China University of Science & Technology Sch Basic Med Sci	6
6	Hou, Huaxin	Guangxi Medical University Coll Pharm	5
7	Tian, Wei	Guangxi Int Zhuang Med Hosp, Nanning 530201	5
8	Kang, Jiankang	Guangxi Medical University	5
9	Chen, Zhipeng	Nanjing University of Chinese Medicine	5

### 3.4 Institutional network analysis

**Figure 4:**
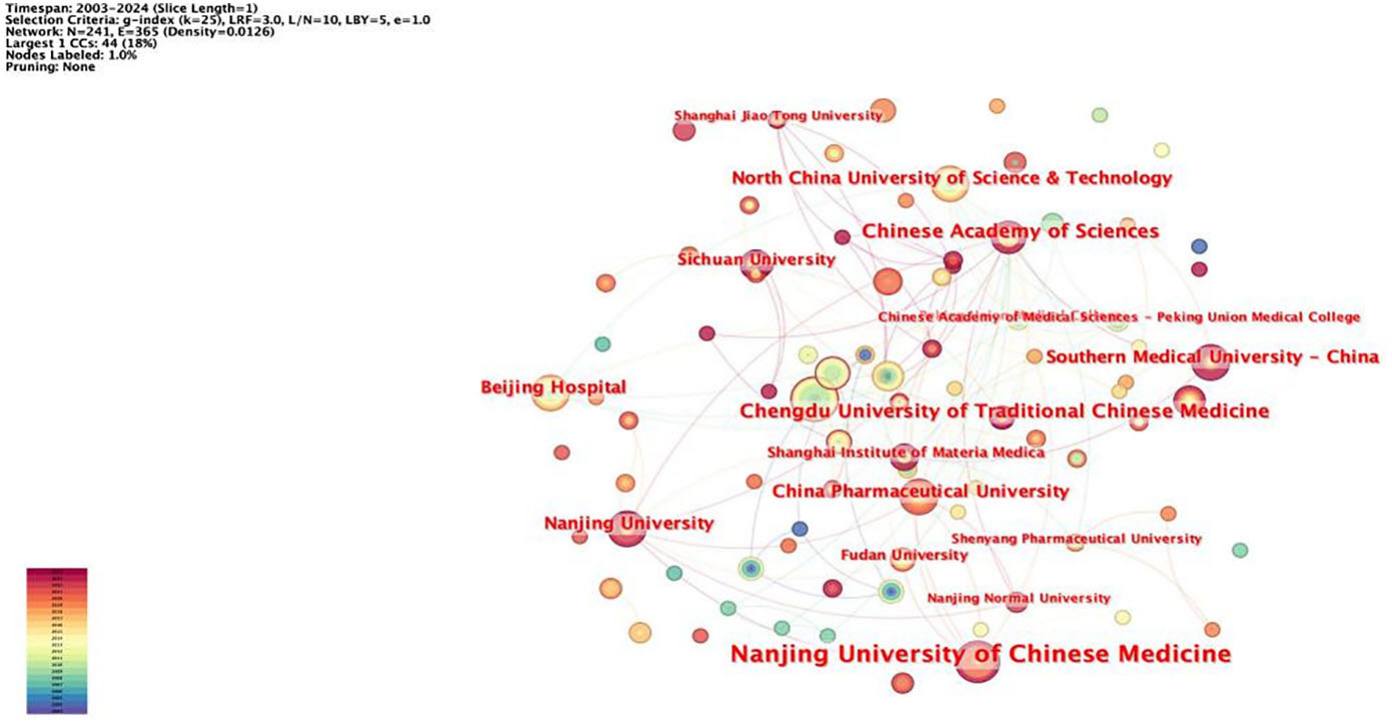
Institutional co-occurrence mapping.

The network node type “Institution” was set, and the network of institutions was mapped (**Figure 4**). The total number of nodes in the network was 476 with 365 lines, and the network density was 0.0126. The statistics for the number of articles published by institutions with more than six articles are shown (**Table 3**). The institutions with the highest number of published papers include Nanjing University of Chinese Medicine (13 papers) and China Medical University Taiwan (13 papers). Subsequently, institutions with a relatively large number of published papers include the China Medical University Hospital Taiwan, Chengdu University of Traditional Chinese Medicine, the Chinese Academy of Sciences, and Southern Medical University. At present, the universities and affiliated hospitals of Chinese medicine are the main institutions for the research of rhein for tumor treatment. Among them, in terms of intermediary centrality, Chengdu University of Traditional Chinese Medicine (0.03) and the Chinese Academy of Sciences (0.04) are larger, which indicates that they have a closer cooperative relationship with other research institutions.

**Table 3:** Volume of institutional communications.

Serial number	Institution	Number of articles
1	NANJING UNIVERSITY OF CHINESE MEDICINE	13
2	CHINA MEDICAL UNIVERSITY TAIWAN	13
3	CHINA MEDICAL UNIVERSITY HOSPITAL TAIWAN	9
4	CHENGDU UNIVERSITY OF TRADITIONAL CHINESE MEDICINE	8
5	CHINESE ACADEMY OF SCIENCES	8
6	SOUTHERN MEDICAL UNIVERSITY CHINA	8
7	ASIA UNIVERSITY TAIWAN	7
8	GUANGXI MEDICAL UNIVERSITY	7
9	BEIJING HOSPITAL	6
10	CHINA PHARMACEUTICAL UNIVERSITY	6
11	NANJING UNIVERSITY	6
12	NORTH CHINA UNIVERSITY OF SCIENCE TECHNOLOGY	6

### 3.5 Country-area analysis

**Figure 5:**
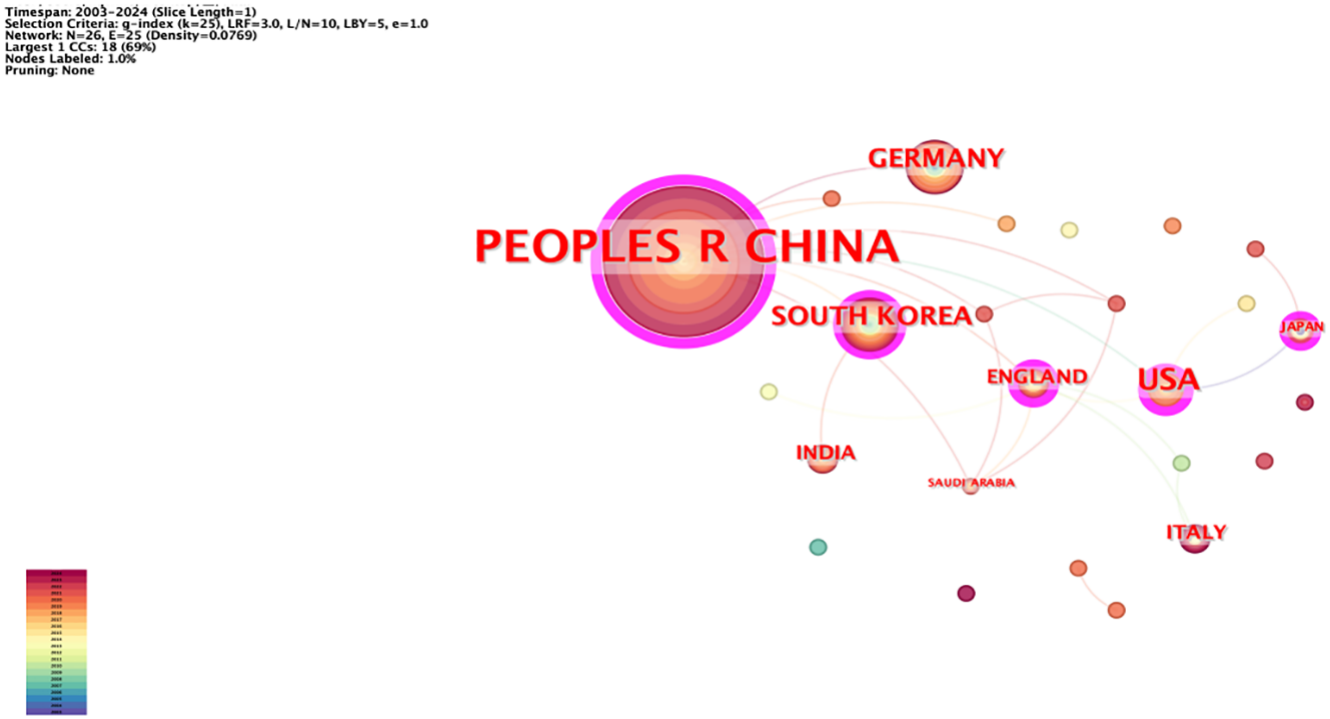
Country co-occurrence mapping.

The network node type “Country” was used to map the country-region network (**Figure 5**). The network had 26 nodes and 25 lines, with a network density of 0.0769, and the authors were distributed across 26 countries (regions). The top five in terms of number of posts were China (146 articles), United States (22 articles), Germany (13 articles), South Korea (13 articles), and United Kingdom (7 articles). In terms of intermediary centrality, China (0.56) has a more extensive cooperation with other countries.

### 3.6 Keyword co-occurrence analysis

**Figure 6:**
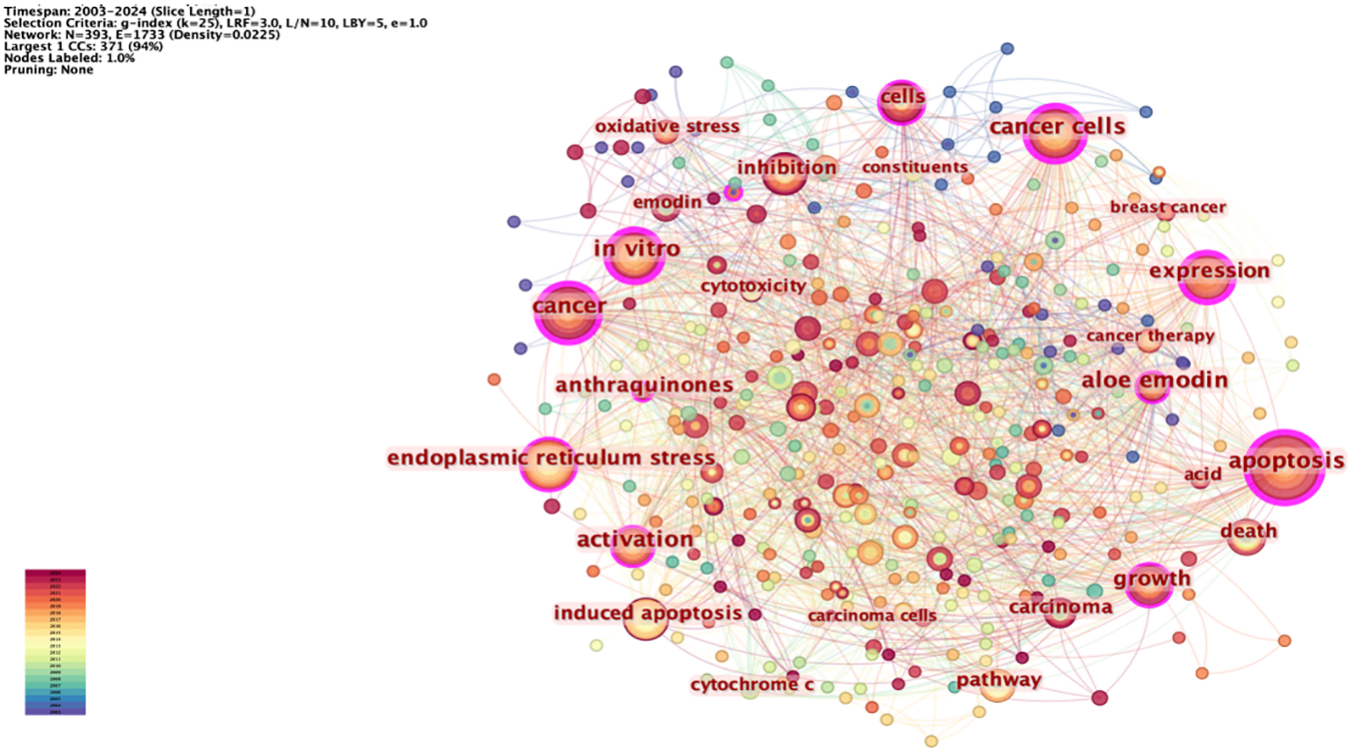
Keyword co-occurrence mapping.

Keywords can summarize the themes and core content of the literature research. The network node type “Keyword” was set, and the keyword co-occurrence map was drawn (**Figure 6**). The network has 393 nodes and 1,733 lines, and the network density value is 0.0225; through the co-occurrence analysis of the keywords, we can understand the hotspots of research in this field. The hot keywords of this mapping include apoptosis, tumor, endoplasmic reticulum stress, inhibition, expression, and pathway. This shows that rhein can exert antitumor effects through the pathways mentioned above, and the research hotspots also focus on the study of its antitumor mechanism.

### 3.7 Keyword clustering analysis

**Figure 7:**
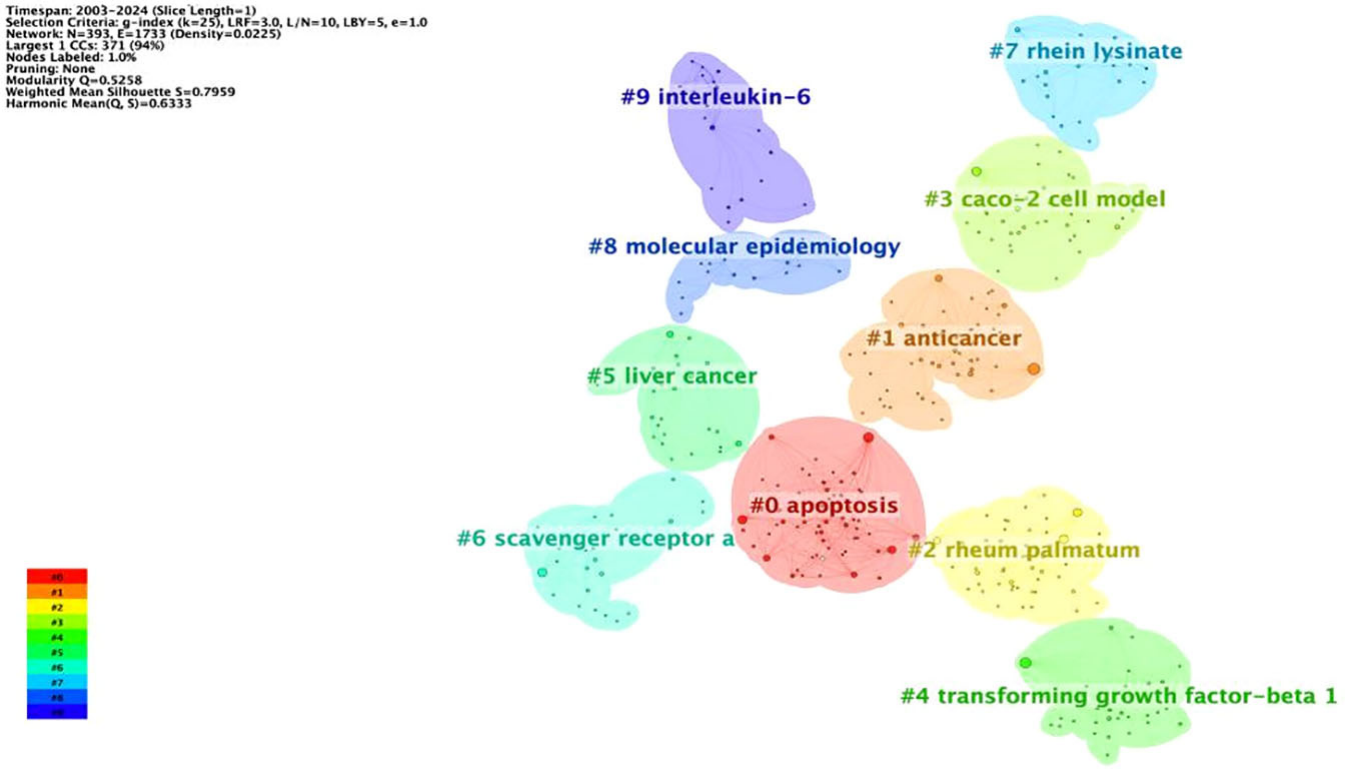
Keyword clustering map.

Because there are too many nodes in the keyword co-linear mapping, keyword clustering is performed, which can classify the categories based on the similarity between keywords and achieve classification to obtain the main research direction of the herbal ingredient rhein and tumors. By plotting the keyword clustering map (**Figure 7**), 10 clusters from #0 to #9 are shown. It is generally accepted that the network clustering module value Q >0.3 indicates a significant clustering structure; that the cluster average profile value S >0.5 clustering is reasonable; and that S >0.7 clustering is convincing. The network had a Q value of 0.5258 and an S value of 0.7959, indicating a significant clustering structure. From the CiteSpace clustering analysis, the keywords can be categorized into the following categories: promotion of apoptosis, anticancer activity, CACO-2 cell model, transforming growth factor β1 (TGF-β1), lysine salt of rhein, molecular epidemiology, interleukin-6 and so on. This indicates that rhein’s research direction for tumor treatment focuses on its mechanism of action. In addition, rhein’s poor water solubility limits its clinical applications to a large extent. Many researchers have attempted to enhance its solubility in water by structural modification of its No. 2 carboxyl, No. 4, and No. 5 hydroxyl groups, and to synthesize a variety of rhein derivatives to improve its bioavailability and antitumor activity (38–41). He et al. designed and synthesized new rhein-piperazine-furanone hetero compounds as raw materials, which were effective in the treatment of cytotoxicity and selectivity in A549 lung cancer cells were significantly enhanced (42). Chen et al. synthesized a rhein amide derivative by connecting rhein to methyl 6-aminohexanoate through an amide bond, and this derivative had a significant inhibitory effect on glioblastoma (43). Tan et al. synthesized a compound by connecting cisplatin and rhein through an ester bond, which was superior to cisplatin in the inhibition of human lung cancer, showed no toxicity to normal human cells, and showed a high degree of toxicity. This compound has a better inhibitory effect on human lung cancer than cisplatin and is non-toxic to normal human cells; therefore, it is highly safe (44).

### 3.8 Keywords with citation bursts

**Figure 8:**
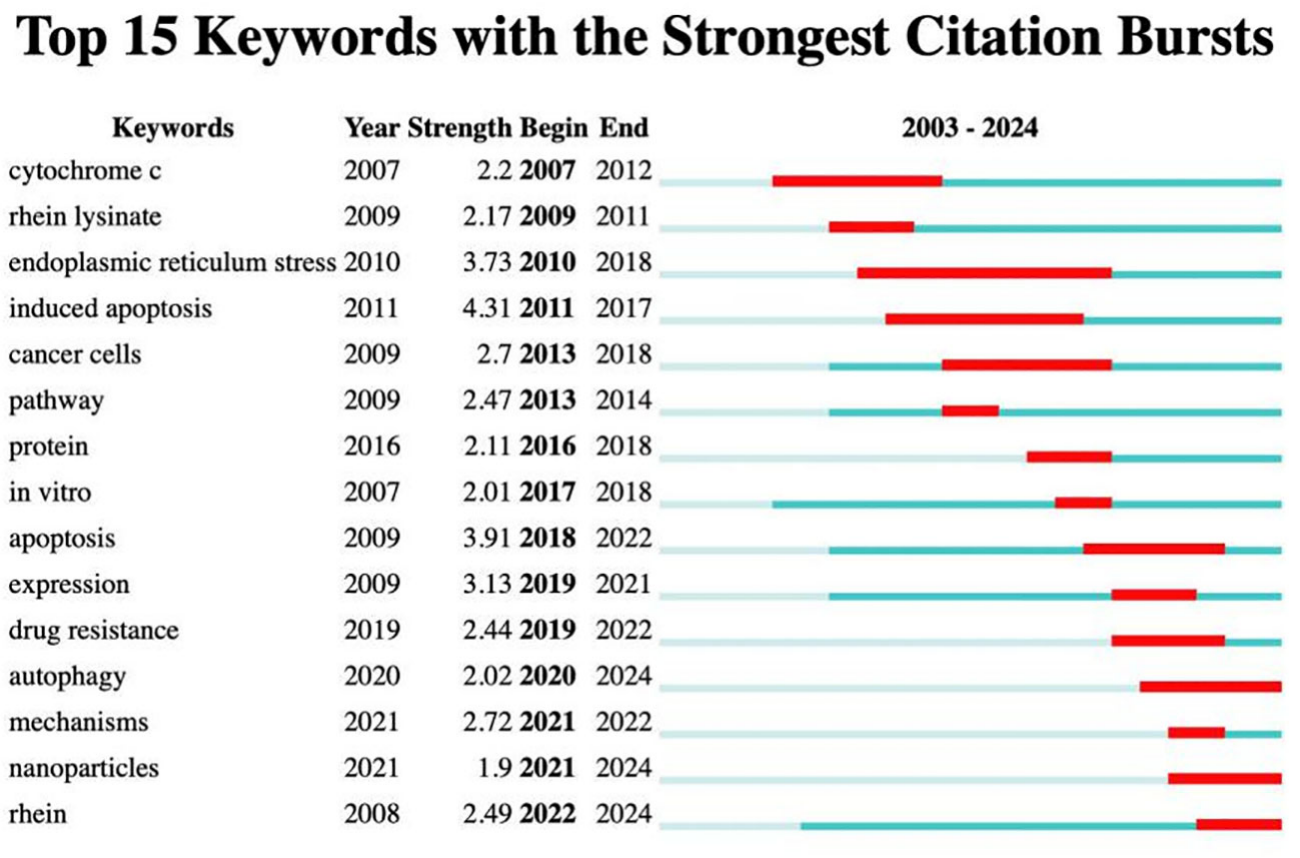
Keyword burstness analysis.

Burst keywords were considered to be indicators of emerging trends, indicating that the term received special attention from scholars during this period. The use of burst analysis can effectively identify emerging dynamic concepts and potential research topics in a particular subject area, which is particularly suitable for tracking new trends in academic development, thus revealing current hotspots in the field of scientific research. For the keywords with citation bursts (**Figure 8**), in which the years with no significant change in keywords are indicated in blue and the years with considerable increase are indicated by red line segments, a total of 15 emergent words were obtained. By analyzing the intensity of the emergent terms, it was found that “inducted apoptosis” (4.31) and “endoplasmic reticulum stress” (3.73) were in the top intensity, which was a hotspot in the research of the antitumor mechanism of rhein and has been discussed for a long time. “Drug resistance,” “autophagy,” and “nanoparticles” were prominent words that started to appear in 2019. It has been found that rhein, in combination with certain chemotherapeutic drugs, can reverse drug resistance and increase the efficacy of radiotherapy. In addition, nanotechnology-based development of nano-drug-carrying systems that can be loaded with rhein to improve its solubility and bioavailability whereas targeting tumors will be a hotspot and a direction of research at this stage and in the future (45–47). Zhang et al. prepared a rhein-embedded docetaxel nanopolymer. They combined it with acoustic dynamics for the treatment of lung cancer, which increased the solubility and cellular uptake of doxycycline and was characterized by fast drug release, good stability, and a better synergistic antitumor effect (48).

## 4 Discussion

Tumors remain a major public health problem worldwide, and the incidence of cancer is still on the rise, seriously affecting human health and quality of life. Rhein is an anthraquinone component widely found in Chinese herbal medicine, which has anti-inflammatory, bactericidal, laxative, and diuretic effects. It also has antitumor impacts by promoting apoptosis, inhibiting cell proliferation, invasion, and metastasis, and combining with radiotherapy drugs to reduce toxicity and increase efficacy.

We aimed to perform a bibliometric analysis of rhein in cancer treatment over the past 20 years to present an overview of global trends and provide a new perspective on future research directions.

A total of 220 articles were retrieved from the core collection database. The annual number of papers steadily increased during this period. This suggests that the research interest in this aspect is increasing. Notably, a decline was observed in 2021. One possible reason for this could be the global COVID-19 pandemic that erupted in 2020 and influenced the global healthcare system and research activities.

From author mapping, low author centrality indicates that there is less collaboration between authors. In this study, this implies that there may be a lack of synergy in research efforts, potentially resulting in duplicated work or slower progress in fully understanding the potential of rhein in tumor treatment. In the future, increased collaboration among authors could lead to more comprehensive and in-depth research. For example, researchers could combine their different expertise and experimental approaches to explore new aspects of rhein’s antitumor mechanisms.

The institutions with the highest number of articles included Nanjing University of Chinese Medicine and China Medical University (Taiwan). China has published the most articles in this field. As the country with the largest number of publications, China may benefit from its extensive historical background, abundant medical resources, unique theoretical systems, and robust scientific research support. In the future, China should strengthen cooperation with the international community to expand the research scope and perspectives.

Keyword burst analysis showed that prominent terms associated with antitumor properties of rhein include induced apoptosis, endoplasmic reticulum stress, inhibition of proliferation, drug resistance, and nanoparticles. However, the unique rigid structure of the tricyclic skeleton of rhein leads to poor water solubility and low bioavailability, limiting its clinical application. Many scholars consider rhein as a lead compound and try to structurally modify its moiety by introducing amino acid fragments, such as lysine and glycine, which significantly enhance the water solubility of rhein, and its antitumor activity is more prominent. When CiteSpace software conducts keyword burst analysis, the latest burst word is “nanoparticle”. Combining polymer with rhein can form stable nanoparticles, which can enhance tissue targeting, improve *in vivo* stability and bioavailability, and thus enhance antitumor activity. Various nanocarrier systems have been developed in the field of oncology. By improving the delivery and bioavailability of rhein through nanotechnology, it holds great promise for overcoming the limitations of its poor solubility. Future research should focus on optimizing these nanoparticle formulations to ensure their safety and efficacy in clinical settings. Additionally, further studies are needed to explore the long-term effects and potential side effects of rhein and its derivatives, as well as their interactions with other drugs commonly used in cancer treatment.

## 5 Conclusions

In summary, this study analyzed the current status and development trend of rhein research for the treatment of tumors over the last two decades. The antitumor mechanism of rhein has been a hot spot of concern, and the preparation of nanoparticles by modification of its chemical groups to improve its bioavailability and targeting and to enhance its antitumor activity will be the frontiers of future research.

## Data Availability

The raw data supporting the conclusions of this article will be made available by the authors, without undue reservation.
